# Metagenome-assembled genomes of acetogenic enrichment obtained from deep subsurface archean granitoids of Koyna Seismogenic Zone, Koyna, India

**DOI:** 10.1128/mra.00922-24

**Published:** 2025-02-25

**Authors:** Rajendra Prasad Sahu, Pinaki Sar

**Affiliations:** 1 Department of Bioscience and Biotechnology, Indian Institute of Technology Kharagpur, Kharagpur, West Bengal, India; Montana State University, Bozeman, Montana, USA

**Keywords:** acetogenic bacteria, terrestrial deep biosphere, Koyna Seismogenic Zone, metagenome-assembled genome, archean granitoids

## Abstract

We report 14 metagenome-assembled genomes (MAGs) of acetogenic bacteria (acetogens) enriched from deep (1,679–2,912 m below surface), hot (55°C–74 °C) granitoids of the Koyna Seismogenic Zone, India. These MAGs include *Thermoanaerobacter pseudethanolicus, Exiguobacterium alkaliphilum, Moorella humiferrea, Caldanaerobacter subterraneus*, etc. The study allowed access to the genomes of deep biosphere acetogens.

## ANNOUNCEMENT

Acetogens are important members of energy-starved deep biosphere (1). Despite their critical ecological roles in driving primary production in deep biosphere through the Wood–Ljungdahl pathway (2) and biotechnological potential in C1 gas valorization (3), the genomes of these organisms remain understudied. Here, we present 14 bacterial MAGs enriched from deep, hot, Archean basement granitoids.

Seven rock cores spanning a depth range of 1,679–2,912 m and temperature gradient 50°C–74°C were sampled through 3,000 m deep Koyna pilot-borehole (Koyna seismogenic zone, Koyna, 17°17′57.27″ N, 73°44′19.07″ E), India. Standard procedures were followed to minimize samples’ contamination (4, 5). Details of drilling, contamination-free rock sample collection, etc. were mentioned elsewhere (6, 7). Acetogens were enriched by adding rock powder into acetogenic medium (2 g/100 mL) in serum vial supplemented with H_2_ + CO_2_ (80:20 v/v) in the headspace (8). An A35 Don Whitely anaerobic workstation was used. Considering the *in situ* condition, three different incubation temperatures (50°C, 60°C, and 70°C) were used for the enrichment (details of the sampling depth, *in situ* and incubation temperatures of rock samples are provided as Table S1 at https://doi.org/10.6084/m9.figshare.28215026).

Following 6 months’ incubation, 10 mL culture from each set was centrifuged (14,000×*g*, 30 min), cell pellet was used for total DNA extraction using QIAGEN Powersoil DNA isolation kit. Pair-end library was synthesized using Illumina DNA Prep, (M) Tagmentation kit followed by shotgun metagenome sequencing using Illumina NovaSeq 6000 (2 × 101 bp chemistry). Default parameters were used for sequencing and all software unless otherwise stated. Sequenced reads were demultiplexed (Illumina bcl2fastq [2.20]) and following adaptor removal (Skewer v0.2.2 [9]), their quality was analyzed (FastQC v0.12.1 [10]) and trimmed (Trimmomatic v0.39 [11]). Human contamination reads were removed (bowtie2 v2.5.3 together with samtools v1.7 and human host genome GRCh38 bowtie2 database [12, 13]). Quality filtered reads were assembled using MEGAHIT-1.2.9 (14), followed by binning through METABAT2 v1.2.9 (15), CONCOCT v1.1.0 (16), and MaxBin2 v2.2.7 (17). Resulting bins were consolidated and refined using Bin_refinement module (metaWRAP v1.3 [18]). GC%, completeness, and contamination percentage were determined (checkM v1.2.2 [19]), followed by genome coverage calculation (quant_bins module of metaWRAP v1.3 [18]). High-quality bins [>90% completeness, <5% contamination, total contigs < 500, N50 >20 kb [20, 21]) were taxonomically affiliated through GTDB-Tk v2.1.1 (22), and resulting alignments were used to build phylogenetic tree using FigTree v1.4.4 (http://tree.bio.ed.ac.uk/software/figtree/). Bins were annotated with PGAP v6.7 and kb_DRAM v0.1.2 (23, 24).

**Fig 1:**
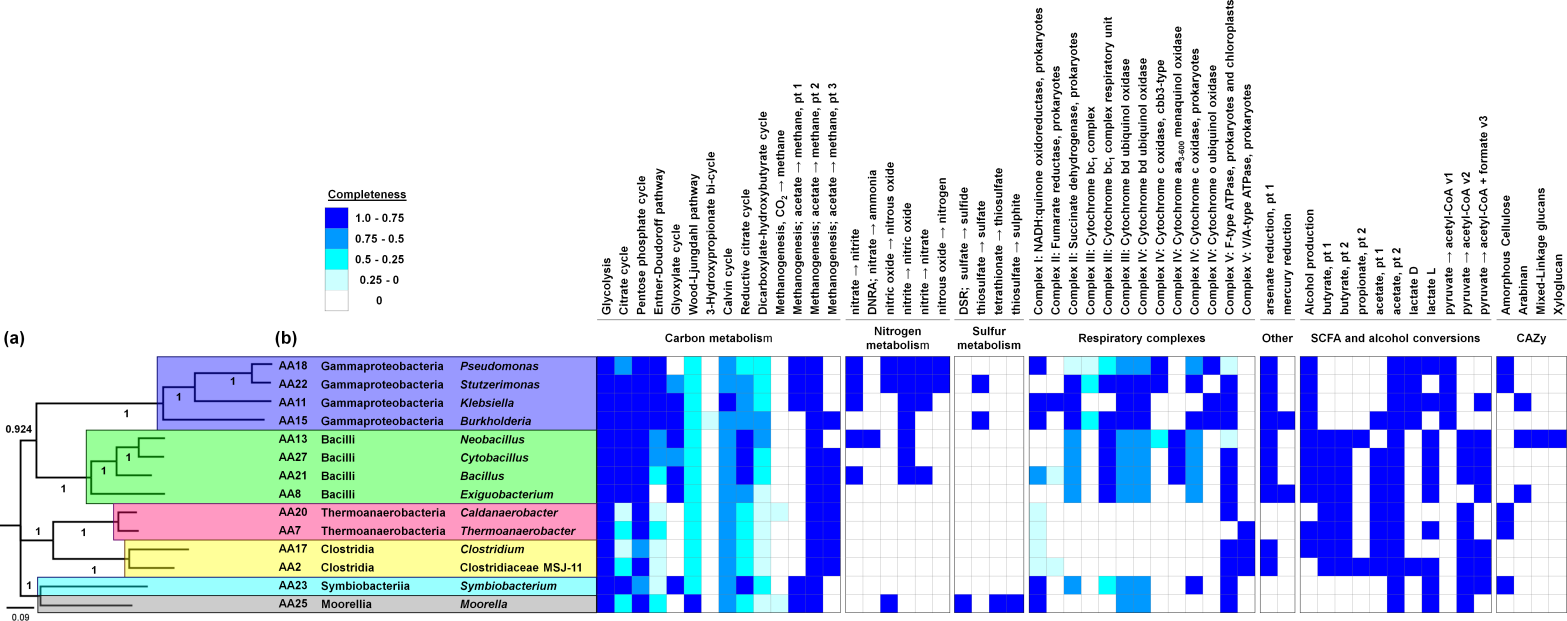
(a) Maximum likelihood tree of 14 metagenome-assembled genomes (MAGs). The phylogenetic tree was constructed using a concatenated set of 120 conserved bacterial marker genes from each of these MAGs using GTDB-Tk “classify” function and FigTree v1.4.4.. (b) Heatmap displays the pathways fully or partially present in each MAG, as predicted by kb_DRAM (24).

During this study 113.62 million quality filtered reads were assembled into 29,442 contigs. These were binned into 14 high-quality MAGs with average size and GC content 4.25 Mb and 48.06%, respectively (Table 1). MAGs consisted of 33–365 contigs (average N50 value 108.57 kb) with coverage varied between 3.84x and 799.97x. Annotation of MAGs showed presence of 2,323–7,845 number of coding DNA sequences (CDS). Taxonomic diversity and functional potential of the MAGs are presented in Fig. 1.

**TABLE 1:** Detailed taxonomy, GeneBank accession numbers, and statistics of 14 metagenome-assembled genomes (MAGs) obtained from acetogenic enrichment of deep subsurface Archean granitoids present underneath the Deccan Traps, India

Mag id	Taxonomy (as per GTDBtk)	GeneBank accession	Completeness (%)	Contamination (%)	Genome coverage (x)	GC (%)	Size (Mb)	No. of contig	N50 (Mb)	Total CDS	RNA
AA2	Bacteria; Bacillota; Clostridia; Clostridiales; Clostridiaceae; MSJ-11	JBFNFA000000000	100	1.61	177.34	30.3	4.06	33	0.27	3,990	43
^*a*^AA7	Bacteria; Bacillota; Thermoanaerobacteria; Thermoanaerobacterales; Thermoanaerobacteraceae; *Thermoanaerobacter*; *Thermoanaerobacter pseudethanolicus*	JBFNEX000000000	97.6	0	799.97	34.4	2.26	63	0.05	2,323	58
AA8	Bacteria; Bacillota; Bacilli; Exiguobacterales; Exiguobacteraceae; *Exiguobacterium*; *Exiguobacterium alkaliphilum*	JBFNEX000000000	98.34	0	164.86	52.8	2.9	48	0.11	2,950	31
AA11	Bacteria; Pseudomonadota; Gammaproteobacteria; Enterobacterales; Enterobacteriaceae; *Klebsiella*; *Klebsiella aerogenes*	JBFNET000000000	99.37	0.27	4.1	55.2	4.89	154	0.06	4,671	46
AA13	Bacteria; Bacillota; Bacilli; Bacillales; DSM-18226; *Neobacillus*	JBFNER000000000	91.81	3.38	11.06	38.7	4.8	87	0.11	4,686	33
AA15	Bacteria; Pseudomonadota; Gammaproteobacteria; Burkholderiales; Burkholderiaceae; *Burkholderia*; *Burkholderia contaminans*	JBFNEP000000000	100	0.42	20.16	66.2	8.66	83	0.17	7,845	59
^*a*^AA17	Bacteria; Bacillota; Clostridia; Clostridiales; Clostridiaceae; *Clostridium*; *Clostridium* sp002453475	JBFNEN000000000	98.46	1.9	8.6	30	3.67	65	0.09	3,451	37
AA18	Bacteria; Pseudomonadota; Gammaproteobacteria; Pseudomonadales; Pseudomonadaceae; *Pseudomonas*; *Pseudomonas aeruginosa*	JBFNEM000000000	90.78	0.71	8	66.9	6.17	365	0.03	6,005	16
^*a*^AA20	Bacteria; Bacillota; Thermoanaerobacteria; Thermoanaerobacterales; Thermoanaerobacteraceae; *Caldanaerobacter*; *Caldanaerobacter subterraneus*	JBFNEK000000000	97.2	1.13	37.85	37.7	2.27	139	0.02	2,341	41
AA21	Bacteria; Bacillota; Bacilli; Bacillales; Bacillaceae; *Bacillus*; *Bacillus anthracis*	JBFNEJ000000000	98.61	3.86	13.63	35.1	5.28	34	0.28	5,447	21
AA22	Bacteria; Pseudomonadota; Gammaproteobacteria; Pseudomonadales; Pseudomonadaceae; *Stutzerimonas*; *Stutzerimonas stutzeri*	JBFNEI000000000	99.53	0.14	23.13	64.1	4.38	74	0.15	4,111	51
AA23	Bacteria; Bacillota; Symbiobacteriia; Symbiobacteriales; Symbiobacteriaceae; *Symbiobacterium*	JBFNEH000000000	98.01	0	4.87	68.4	3.66	145	0.05	3,383	46
^*a*^AA25	Bacteria; Bacillota; Moorellia; Moorellales; Moorellaceae; *Moorella*; *Moorella humiferrea*	JBFNEG000000000	99.48	1.36	3.84	54	2.5	205	0.03	2,739	49
AA27	Bacteria; Bacillota; Bacilli; Bacillales; DSM-18226; *Cytobacillus*; *Cytobacillus gottheilii*	JBFNEE000000000	97.53	1.14	16.77	39.1	4.01	64	0.1	3,901	20

^*a*^ Genera reported as acetogens in AcetoBase2 (25).

These MAGs provide access to the genomes of deep rocky-biosphere acetogens allowing us to gain insights into their significance in functioning and adaptation of deep life.

## Data Availability

Raw sequence reads are available in the NCBI GenBank database under Sequence Read Archive accession numbers SRX24612229 (BioProject PRJNA1021524). Draft genomes are available under Genbank accession numbers PRJNA1121667.
